# Supplementary material for: Single cell RNA sequencing of the adult Drosophila eye reveals distinct clusters and novel marker genes for all major cell types
Source: Commun Biol. 2022 Dec 14;5:1370. doi: 10.1038/s42003-022-04337-1 (PMC9751288; doi:10.1038/s42003-022-04337-1)
Supplement: Supplementary file 2 — Supplementary Information [file 42003_2022_4337_MOESM2_ESM.pdf]

Supplementary Figure 1. Monocle 3 clustering of 1-day, 3-day, and 7-day old adult eyes do not show any clear progression of transcriptome changes.

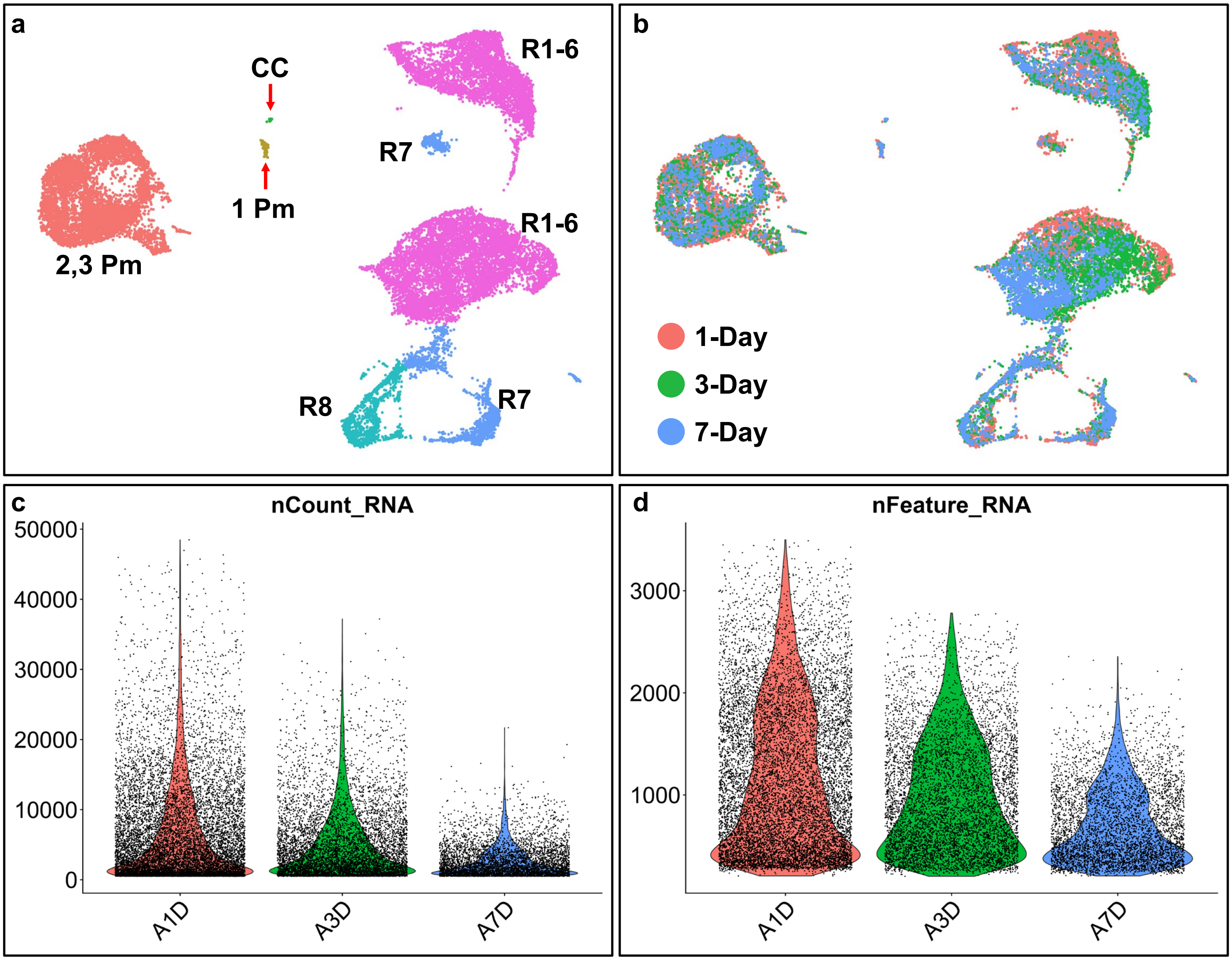

a) Monocle 3 clustering of 1-day, 3-day and 7-day old adult eyes showing the major cell types of the eye. **b**) Same cluster as in **a** but showing the sample age of each cell. All clusters show an intermixing of cells from the three time points. **c**) Violin plot of the number of transcripts per cell (nCount_RNA) in 1-day (A1D), 3-day (A3D), and 7-day (A7D) old male adult eyes. **d**) Violin plot of the number of genes detected per cell (nFeature_RNA) in 1-day (A1D), 3-day (A3D), and 7-day (A7D) old male adult eyes.

Supplementary Figure 2. R8 FeaturePlots for 1-day and 3-day old adult eyes

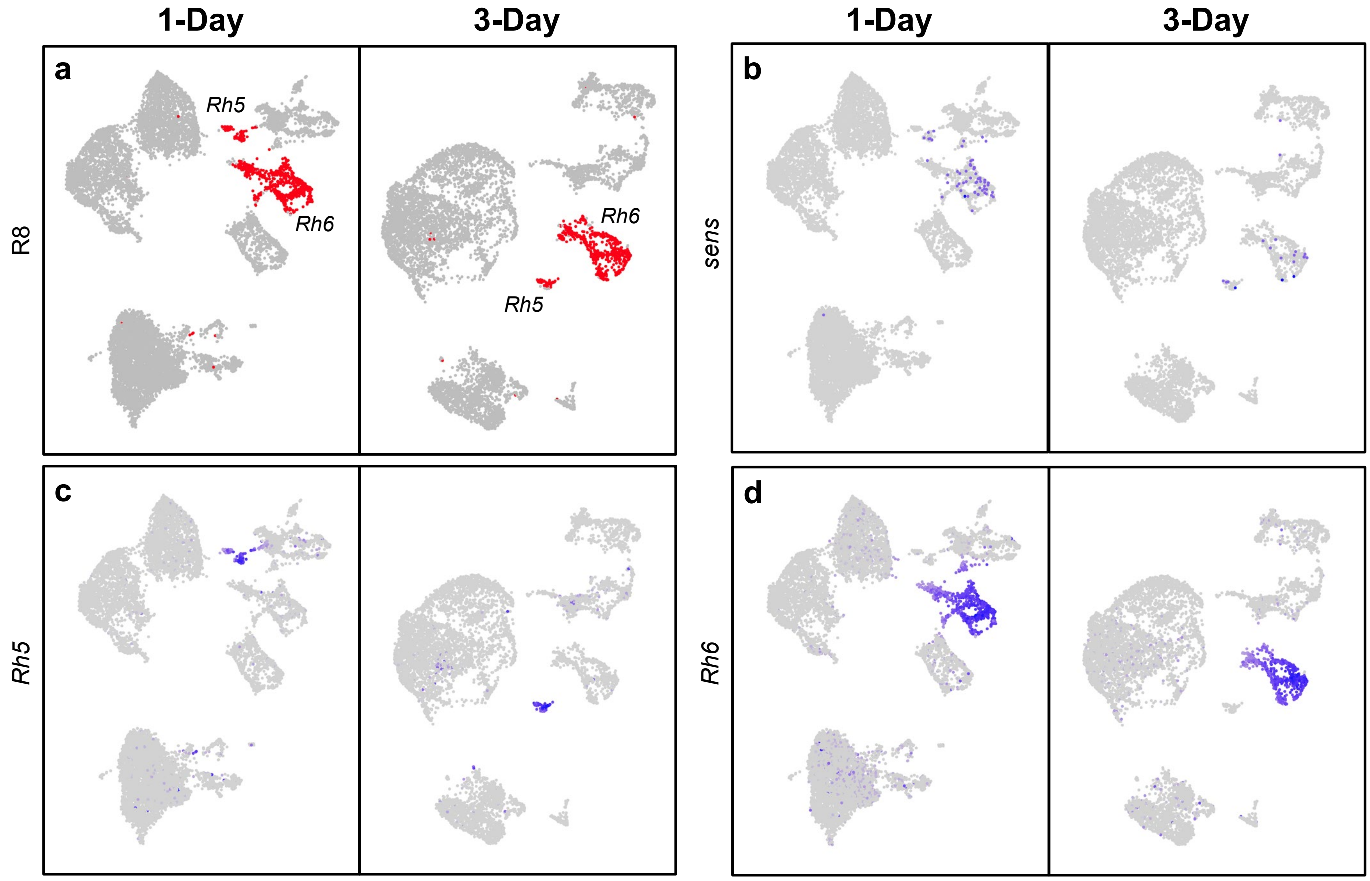

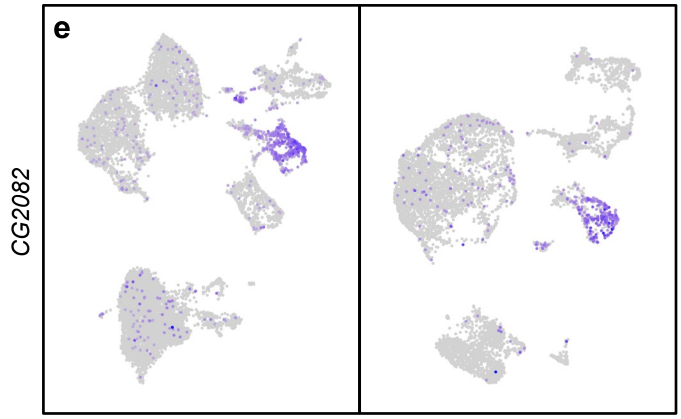

f **Numbers of *Rh5* and *Rh6* R8 cells**

Rh	1-Day	3-Day	7-Day	Expected
Rh5	113 (14%)	58 (10%)	63 (13%)	~30%
Rh6	678 (86%)	517 (90%)	441 (88%)	~70%
**Total**	**791**	**575**	**504**	**100%**

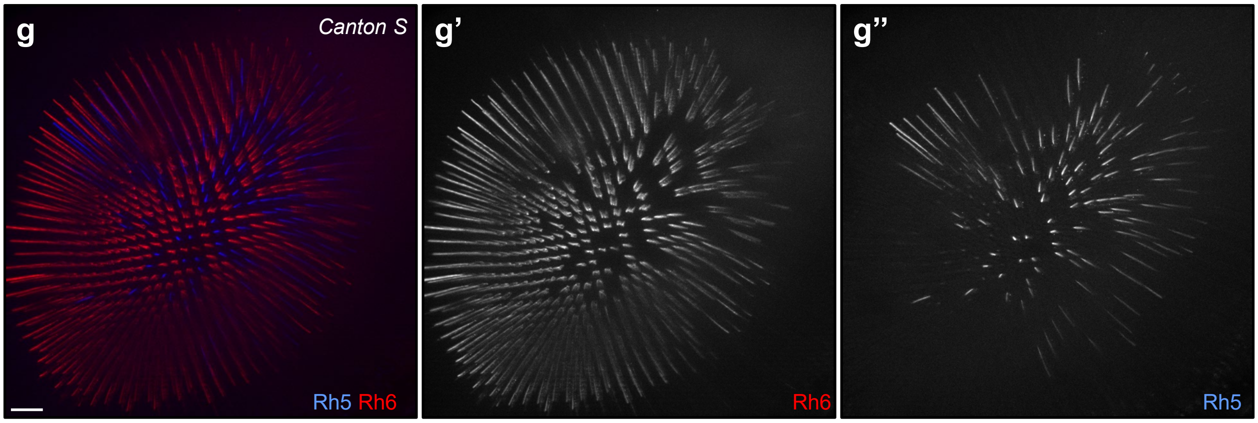

Supplementary Figure 2. R8 FeaturePlots for 1-day and 3-day old adult eyes.

a) Cluster plots showing R8 cells in 1-day and 3-day eye data sets (red). **b-e**) FeaturePlots showing the expression of *sens* (**b**), *Rh5* (**c**), *Rh6* (**d**) and *CG2082* (**e**) in 1-day and 3-day old adult eyes. Cells expressing *sens* and *CG2082* are brought to the front. **f**) Table showing the numbers of *Rh5* and *Rh6* positive R8 cells. **g**) Immunostaining of a *CantonS* eye showing *Rh5* and *Rh6* expression at the expected ~30:70 ratio. Scale bar: 20 μm.

Supplementary Figure 3. R7 FeaturePlots for 1-day and 3-day old adult eyes

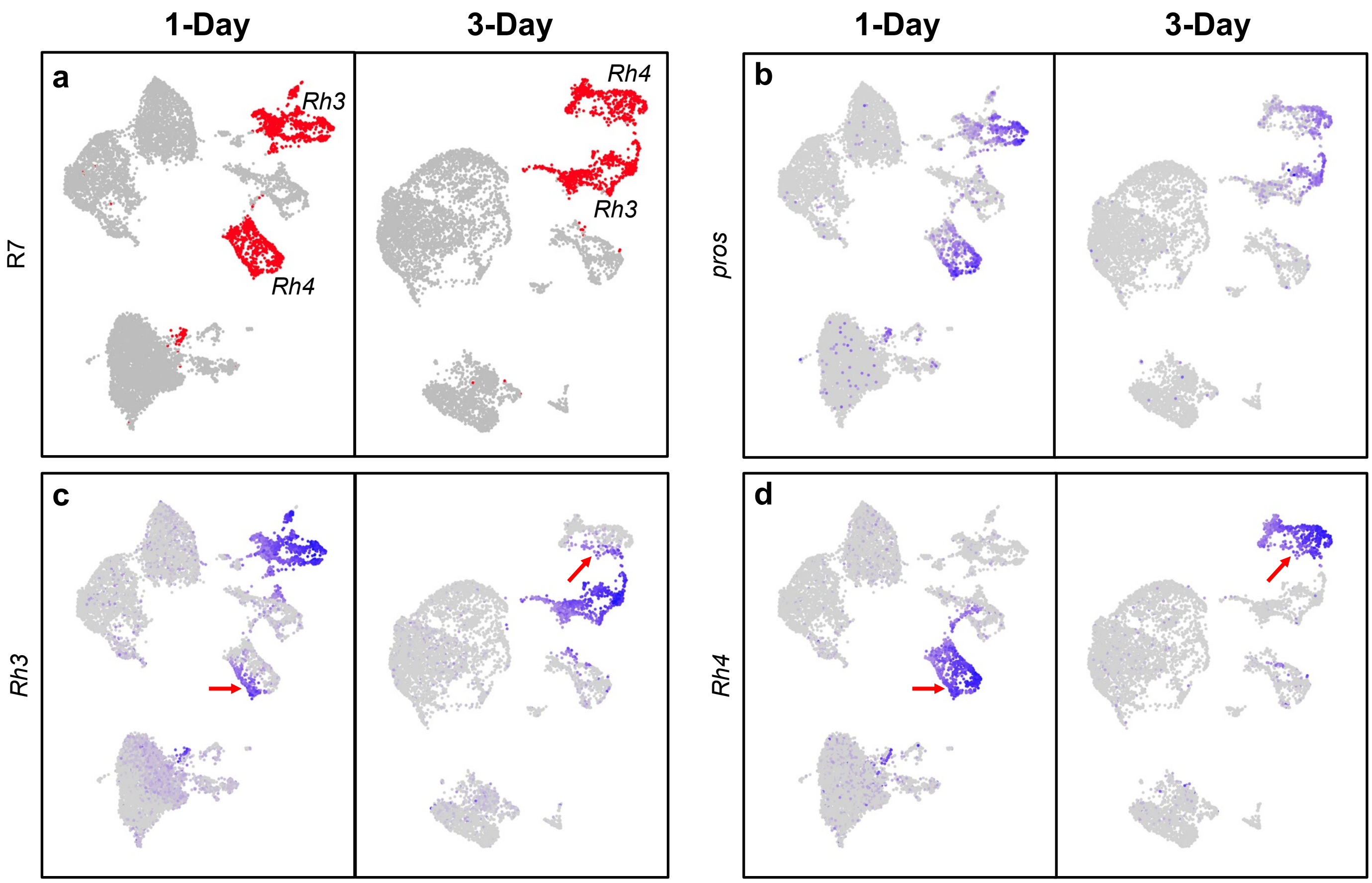

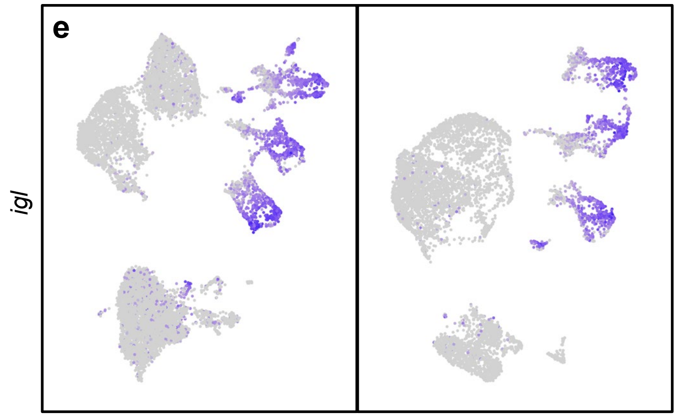

f **Numbers of Pale (*Rh3*) and Yellow (*Rh4*) R7 cells**

Pale / Yellow	1-Day	3-Day	7-Day	Expected
Pale	967 (55%)	666 (53%)	525 (55%)	~30%
Yellow	803 (45%)	585 (47%)	428 (45%)	~70%
**Total**	**1,770**	**1,251**	**953**	**100%**

a) Cluster plots showing R7 cells in 1-day and 3-day eye data sets (red). **b-e**) FeaturePlots showing the expression of *pros* (**b**), *Rh3* (**c**), *Rh4* (**d**) and *igl* (**e**) in 1-day and 3-day old adult eyes. Cells expressing *pros* and *igl* are brought to the front. **f**) Table showing the numbers of *pale* and *yellow* positive R7 cells.

Supplementary Figure 4. Dorsal rim area FeaturePlots for 1-day, 3-day and 7-day old adult eyes

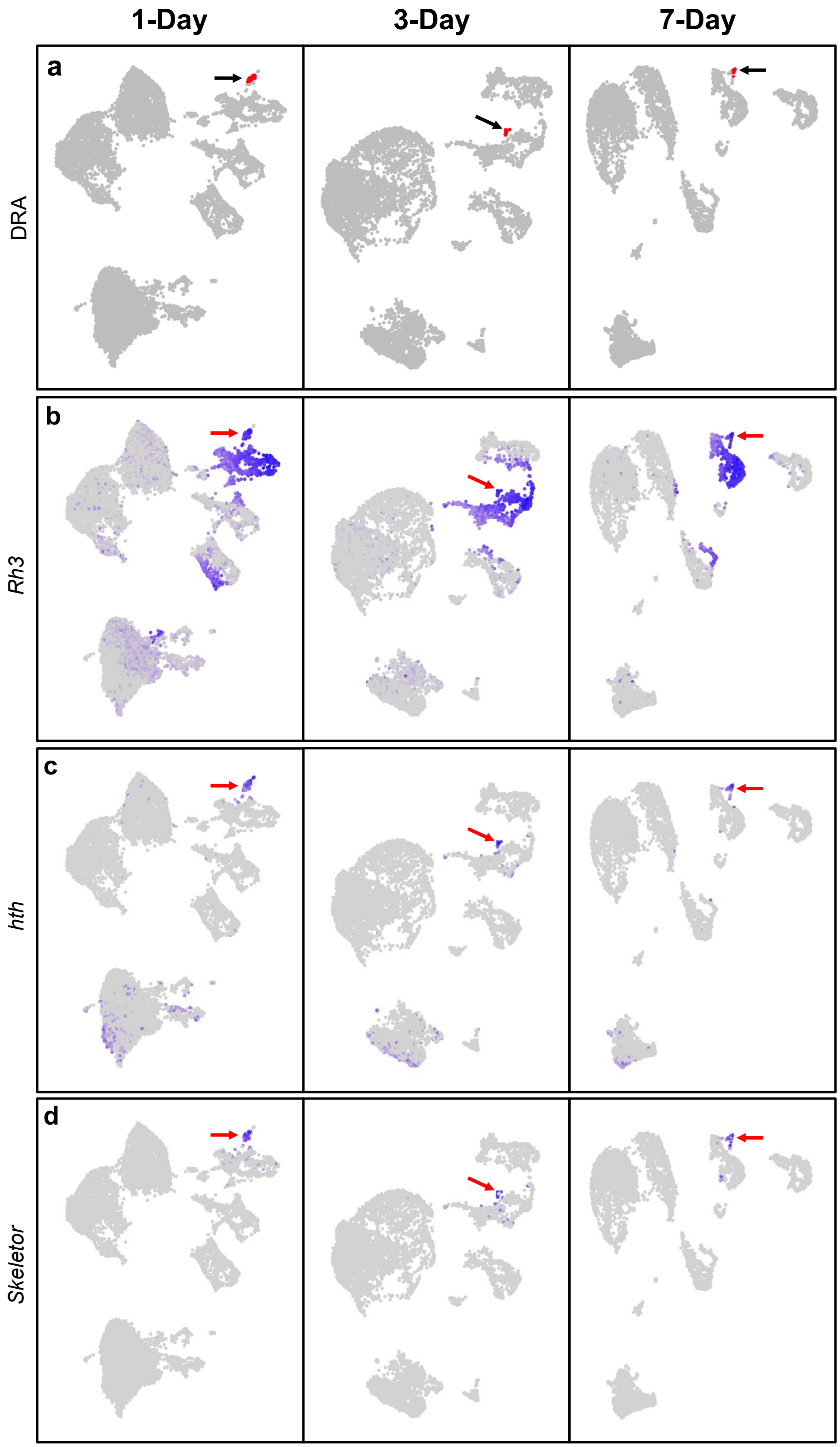

a) Cluster plots showing dorsal rim area R7/R8 cells in 1-day, 3-day and 7-day old eye data sets (red). Black arrow points to the dorsal rim area R7/R8. **b-d**) FeaturePlots showing the expression of *Rh3* (**b**), *hth* (**c**) and *Skeletor* (**d**) in 1-day, 3-day and 7-day old adult eyes.

Supplementary Figure 5. R1-6 cluster and feature plots for 1 day and 3 days old adult eyes

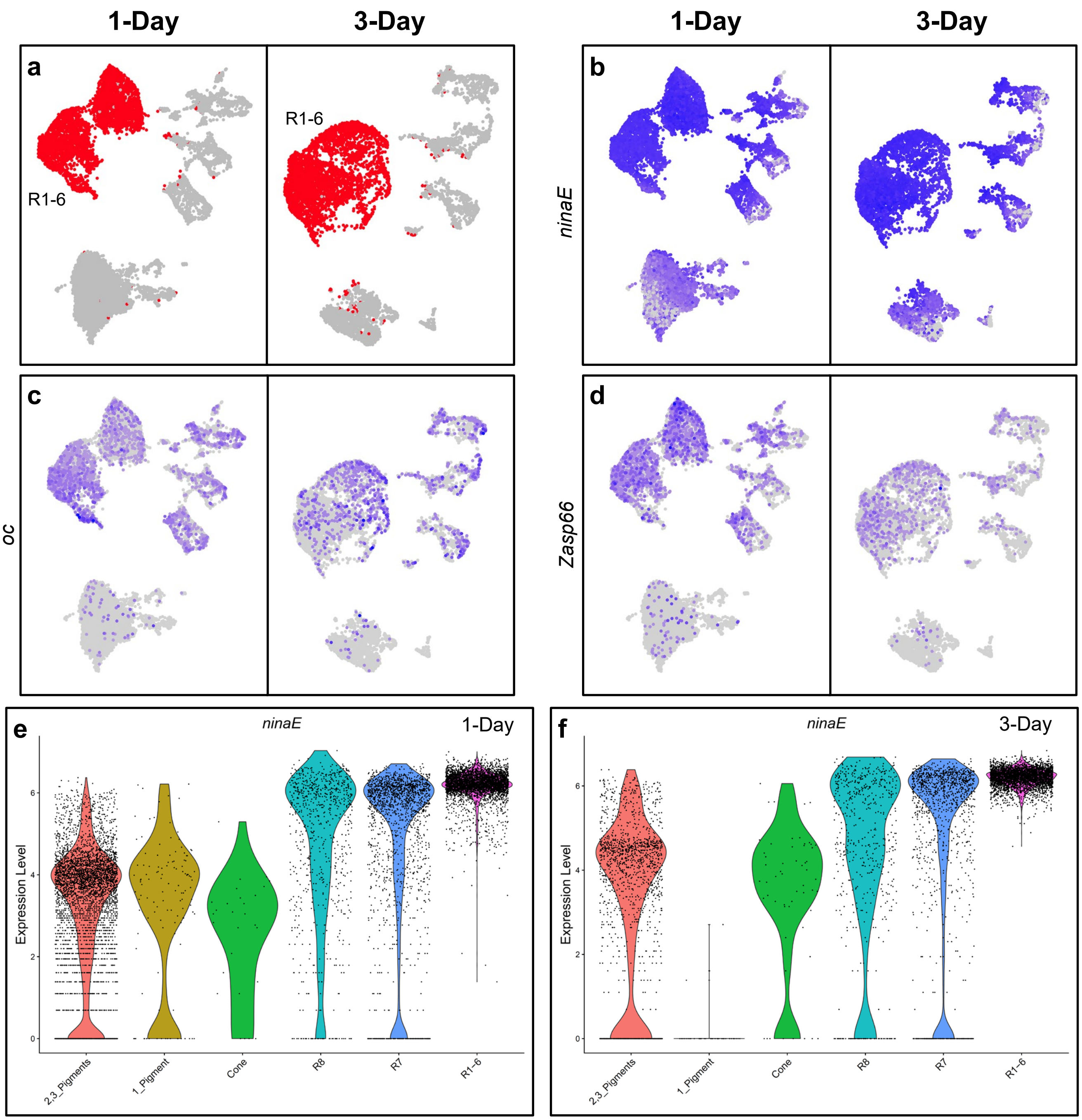

a) Cluster plots showing R1-6 cells in 1-day and 3-day eye data sets (red). **b-d**) FeaturePlots showing the expression of *ninaE* (**b**), *oc* (**c**), and *Zasp66* (**d**) in 1-day and 3-day old adult eyes. Cells expressing *oc* and *Zasp66* are brought to the front. **e**, **f**) Violin plots showing *ninaE* expression in 1-day (**e**) and 3-day (**f**) old adult eyes

Supplementary Figure 6. Cone cell FeaturePlots for 1-day and 3-day old adult eyes

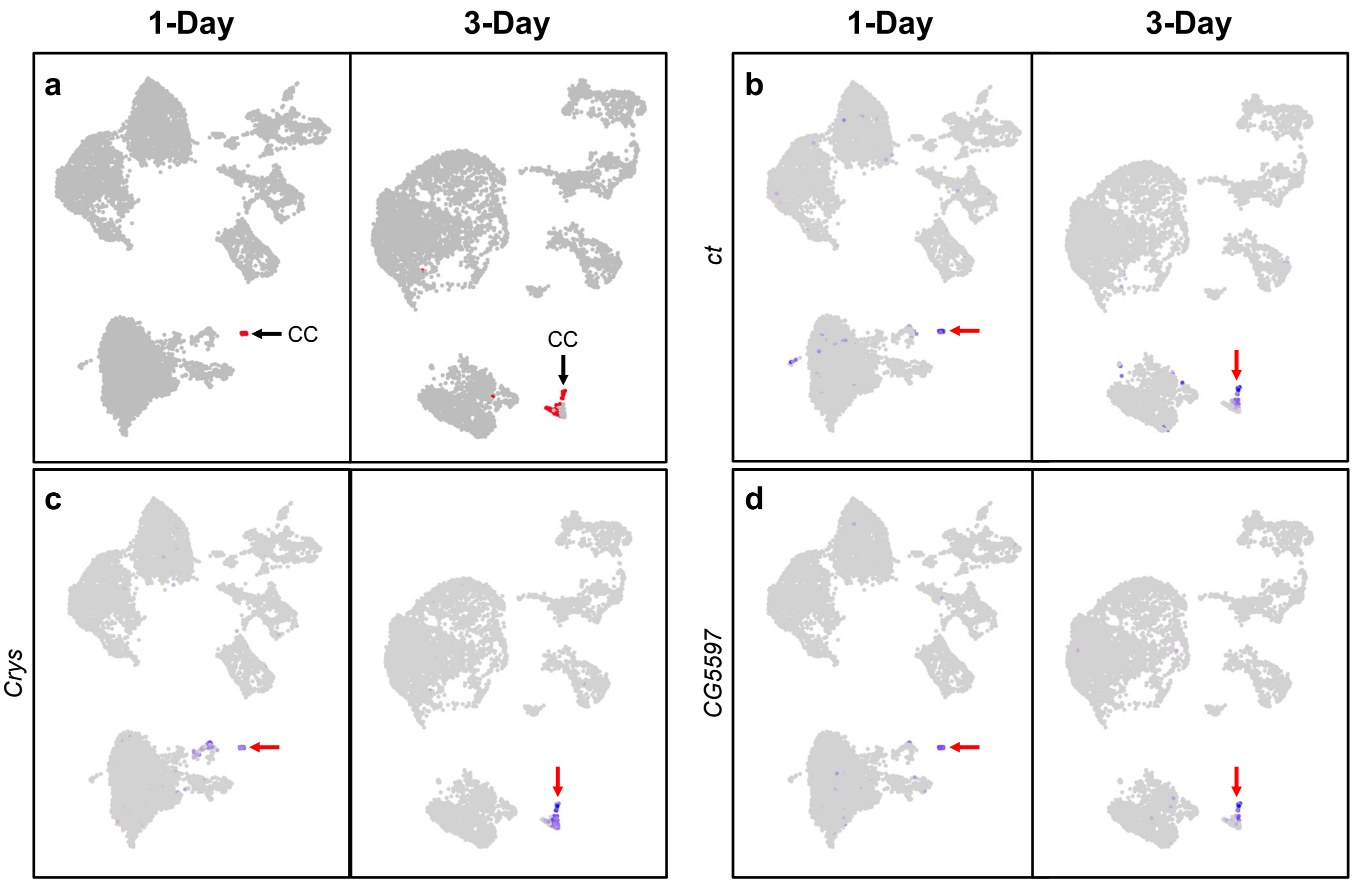

a) Cluster plots showing cone cells in 1-day and 3-day eye data sets (red). Black arrows point to the cone cell clusters. **b-d**) FeaturePlots showing the expression of *ct* (**b**), *Crys* (**c**), *CG5597* (**d**) in 1-day and 3-day old adult eyes. Cells expressing *ct*, *Crys* and *CG5597* are brought to the front.

Supplementary Figure 7. Pigment cell FeaturePlots for 1-day and 3-day old adult eyes

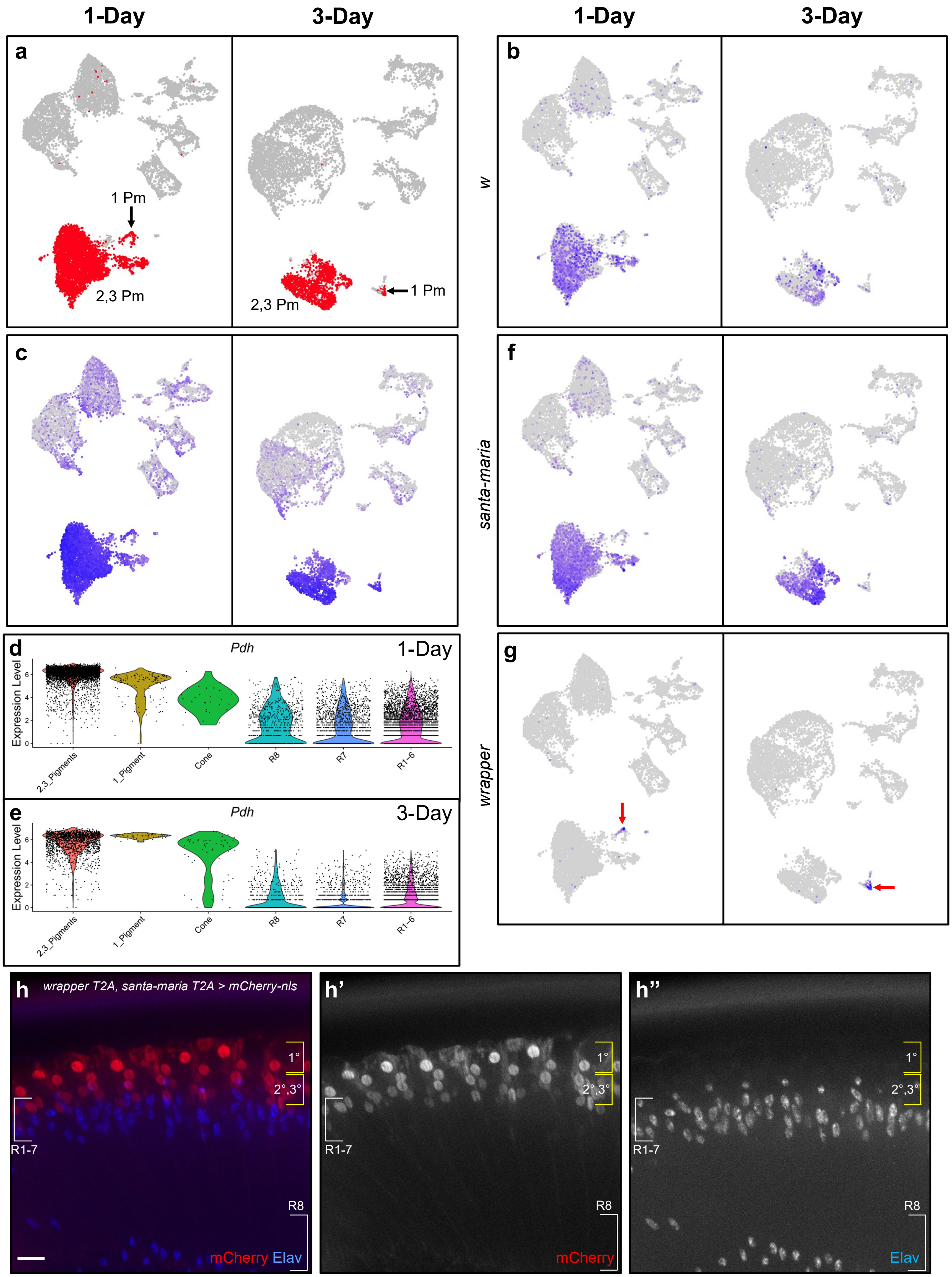

Supplementary Figure 7. Pigment cell FeaturePlots for 1-day and 3-day old adult Eyes.

a) Cluster plots showing pigment cells cells in 1-day and 3-day eye data sets (red). Black arrows point to the smaller primary pigment cell cluster (1 Pm); the remaining red cells are secondary and tertiary pigment cells (2,3 Pm). **b**, **c**) FeaturePlots showing the expression of *w* (**b**) and *Pdh* (**c**) in 1-day and 3-day old adult eyes. **d**, **e**) Violin plots showing *Pdh* expression in 1-day (**d**) and 3-day (**e**) old adult eyes. **f**, **g**) FeaturePlots showing the expression of *santa-maria* (**f**) and *wrapper* (**g**) in 1-day and 3-day old adult eyes. Red arrows point to *wrapper* positive cells in **g**. **h**) Coronal view of a *wrapper-T2A-Gal4, santa-maria-T2A-Gal4* > *UAS-mCherry-nls* adult eye with mCherry stained red and Elav stained blue. Scale bar: 10 μm.

Supplementary Figure 8. *sepia* and *Punch* are expressed in pigment cells

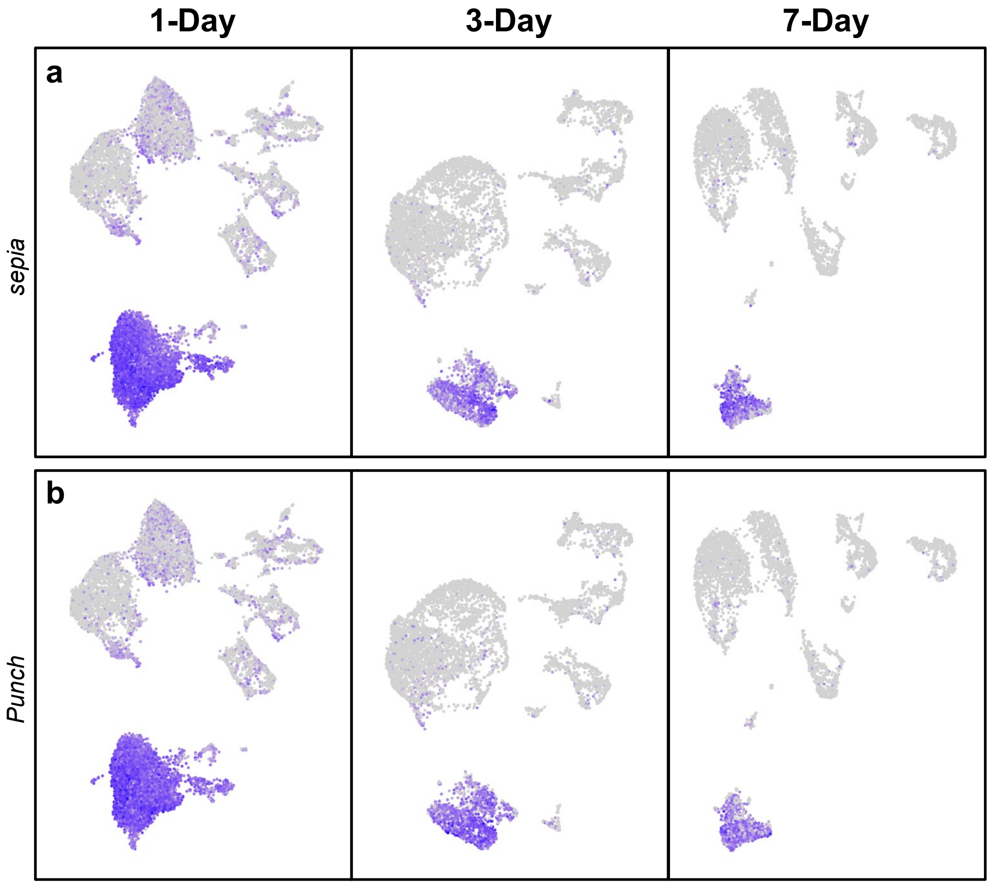

a) FeaturePlots of *sepia* from 1-day, 3-day and 7-day old adult eyes. **b**) FeaturePlots of *Punch* from 1-day, 3-day and 7-day old adult eyes.

Supplementary Figure 9. Validation of dorsal 3rd R7s and dorsal rim area photoreceptors in 1-day adult female eyes

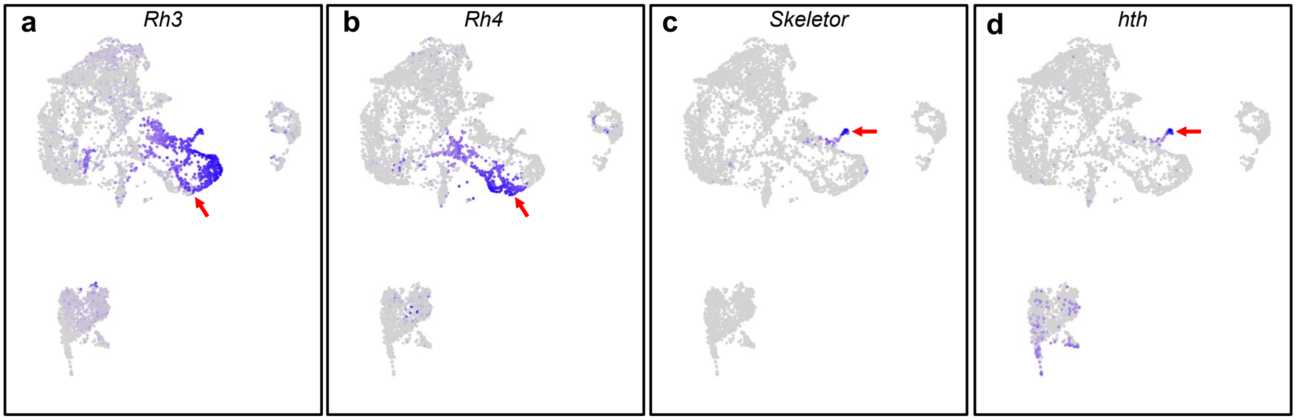

a-d) FeaturePlots of 1-day old adult female eyes showing expression of *Rh3* (**a**), *Rh4* (**b**), *Skeletor* (**c**), and *hth* (**d**). Cells expressing *Skeletor* and *hth* are brought to the front. Red arrows in **a**, **b** point to the dorsal third R7s. Red arrows in **c**, **b** point to dorsal rim area R7/8s.

Supplementary Figure 10. *apolpp* is expressed in most cells in 1-day old male eyes but not in female eyes

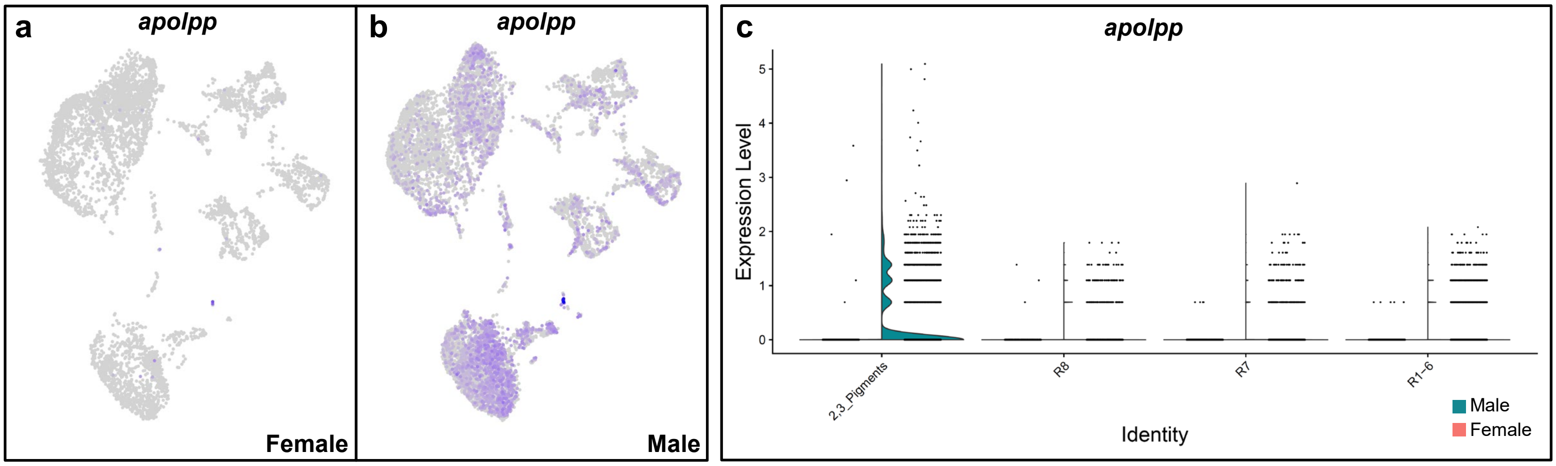

a, b) FeaturePlots showing expression of *apolpp* in 1-day old adult female eyes (**a**) and 1-day old adult male eyes (**b**). All cells expressing *apolpp* are brought to the front. **c**) ViolinPlot showing log normalized *apolpp* expression in male and female eye cells.

Supplementary Figure 11. R7 clustering is not affected by the removal of *Rh5/6* in adult eye clustering

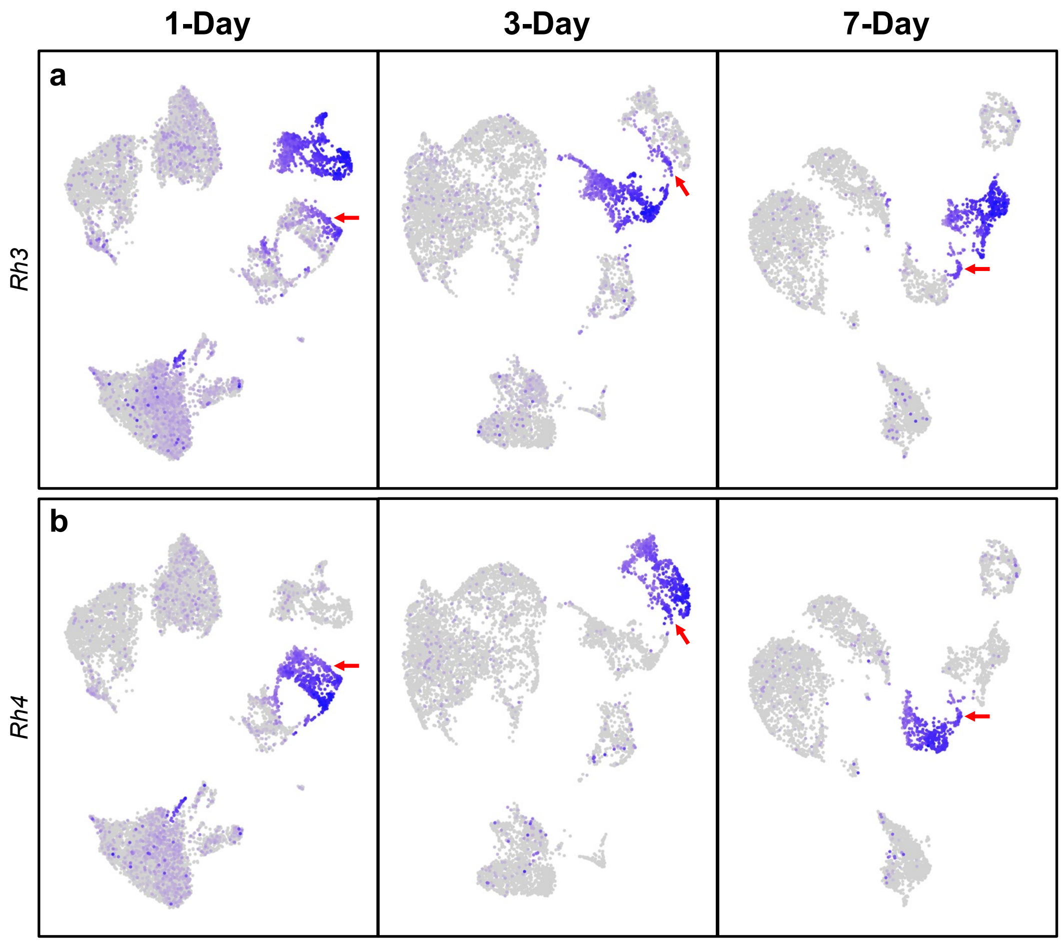

a) FeaturePlots of *Rh3* of 1-day, 3-day and 7-day old adult eye data where *Rh5*/6 count data were removed prior to clustering. **b**) FeaturePlots of *Rh4* of 1-day, 3-day and 7-day old adult eye data where *Rh5*/6 count data were removed prior to clustering. Red arrows point to the dorsal third R7s where Rh3 and Rh4 are co-expressed. Cells expressing Rh3 and/or Rh4 are brought to the front.

Supplementary Figure 12. ***ninaE*** **is not a major contributor to R1-6, R7 nor R8 clustering.**

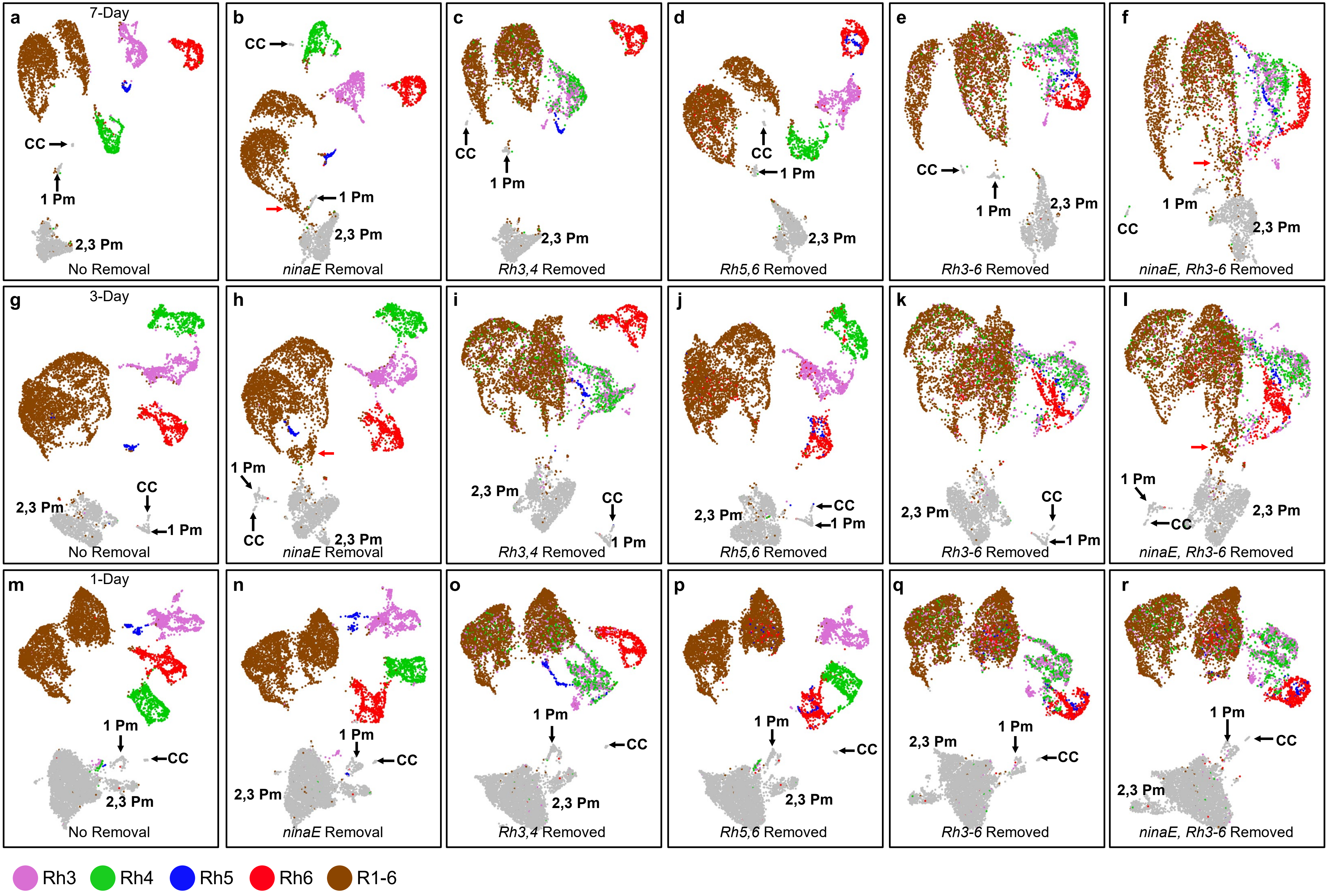

Supplementary Figure 12. ninaE is not a major contributor to clustering of R1-6 cells.
a) UMAP clustering of 7-day old adult eyes where R7 and R8 are shown as four distinct clusters expressing either *Rh3*, *Rh4*, *Rh5*, or *Rh6.* **b-f**) UMAP clustering of 7-day old adult eyes where *ninaE* counts data (**b**), *Rh3/4* counts data (**c**), *Rh5/6* counts data (**d**), *Rh3-6* counts data (**e**), or *ninaE* and *Rh3-6* counts data (**f**) were removed from clustering. **g**) UMAP clustering of 3-day old adult eyes. **h-l**) UMAP clustering of 3-day old adult eyes where *ninaE* counts data (**h**), *Rh3/4* counts data (**i**), *Rh5/6* counts data (**j**), *Rh3-6* counts data (**k**), *ninaE* and *Rh3-6* counts data (**l**) were removed from clustering. **m**) UMAP clustering of 1-day old adult eyes. **n-r**) UMAP clustering of 1-day old adult eyes where *Rh3/4* counts data (**o**), *Rh5/6* counts data (**p**), *Rh3-6* counts data (**q**), or ninaE and Rh3-6 counts data (**r**) were removed from clustering. Only R1-6, R7 and R8s were colored for clarity. Grey cell cluster identities labels: CC are cone cells; 1 Pm are primary pigment cells; 2,3 Pm are secondary and tertiary pigment cells.

Supplementary Table 1. Larval and pupal marker genes expression in adult eyes.

Gene	Expected Larval/Pupal Expression	Adult Eye Expression	Pattern
ato	Furrow, early R8	Not expressed	N
sens	R8	R8	C
boss	Late R8, all photoreceptors in pupae	All cell types	P
ro	R2/5, R3/4	Not expressed	N
svp	R3/4, early R1/6	Not expressed	N
sev	R3/4, R7, cone cells	Not expressed	N
B-H1	R1/6, primary pigment cell, undiff.	2,3 pigment, few cells in all cell types	N
B-H2	R1/6, primary pigment cell, undiff.	Few cells in all cell types	N
pros	R7, cone cells	R7	P
run	Late R8, late R7	In a few R7	N
salm	R3/4, R7, R8, cone cells	R7, R8	P
salr	R3/4, R7, R8, cone cells	R7, R8	P
ct	Cone cells	Cone cells	C
N	R1-8, cone cells	Cone and pigment cells	N
Dl	R1-8, cone cells	Not expressed	N
Ser	Equatorial cells	Not expressed	N
fng	Ventral anterior to furrow cells	Very few pigment cells	N
Egfr	All cells	Cone cells, 2,3 pigment cells	N
spi	R1-8, cone cells	All cell types	P
aos	All photoreceptors, cone cells	2,3 pigment cells	N
pnt	All photoreceptors	Not expressed	N
aop	All cells posterior to furrow except PR precursors	All cell types	C
dpp	Furrow	Some 2,3 pigment cells	N
wg	Polar eye cells	Not expressed	N
hh	R1-7	Not expressed	N
ptc	All cells, strong in furrow	Some 2,3 pigment cells	N
ci	All cells, strong in furrow	Some 2,3 pigment cells	N
gl	All differentiated cells	All cell types	C

Legends

Pattern (C = complete, P = partial and reliable adult cell marker, N = not a reliable marker for same cell types in adult)

undiff. = undifferentiated cells

PR = photoreceptors
