# Supplementary material for: Single cell RNA sequencing of the adult Drosophila eye reveals distinct clusters and novel marker genes for all major cell types
Source: Commun Biol. 2022 Dec 14;5:1370. doi: 10.1038/s42003-022-04337-1 (PMC9751288; doi:10.1038/s42003-022-04337-1)
Supplement: Supplementary file 3 — Description of Additional Supplementary Files [file 42003_2022_4337_MOESM3_ESM.pdf]

Description of Additional Supplementary Files

File name: Supplementary Data 1

Description: All Seurat called markers for R8 marker genes for each male eye time point.

File name: Supplementary Data 2

Description: All Seurat called markers for R7 marker genes for each male eye time point.

File name: Supplementary Data 3

Description: All Seurat called markers for R1-6 marker genes for each male eye time point.

File name: Supplementary Data 4

Description: All Seurat called markers for cone cell marker genes for each male eye time point.

File name: Supplementary Data 5

Description: All Seurat called markers for secondary and tertiary pigment cell marker genes for each male eye time point.

File name: Supplementary Data 6

Description: All Seurat called markers for primary pigment cell marker genes for each male eye time point.

File name: Supplementary Data 7

Description: All Seurat called marker genes for male or female 1 day old eyes.

File name: Supplementary Data 8

Description: Source data for GO Term enrichment shown in Figure 8
