# Supplementary material for: Single cell RNA sequencing of the adult Drosophila eye reveals distinct clusters and novel marker genes for all major cell types
Source: Commun Biol. 2022 Dec 14;5:1370. doi: 10.1038/s42003-022-04337-1 (PMC9751288; doi:10.1038/s42003-022-04337-1)
Supplement: Supplementary file 12 — Reporting Summary [file 42003_2022_4337_MOESM12_ESM.pdf]

Corresponding author(s): Graeme Mardon
Last updated by author(s): Nov 11, 2022

Reporting Summary

Nature Portfolio wishes to improve the reproducibility of the work that we publish. This form provides structure for consistency and transparency in reporting. For further information on Nature Portfolio policies, see our Editorial Policies and the Editorial Policy Checklist.

Statistics

For all statistical analyses, confirm that the following items are present in the figure legend, table legend, main text, or Methods section.

n/a	Confirmed	
☐	☒	The exact sample size (*n*) for each experimental group/condition, given as a discrete number and unit of measurement
☐	☒	A statement on whether measurements were taken from distinct samples or whether the same sample was measured repeatedly
☒	☐	The statistical test(s) used AND whether they are one- or two-sided<br>*Only common tests should be described solely by name; describe more complex techniques in the Methods section.*
☒	☐	A description of all covariates tested
☒	☐	A description of any assumptions or corrections, such as tests of normality and adjustment for multiple comparisons
☒	☐	A full description of the statistical parameters including central tendency (e.g. means) or other basic estimates (e.g. regression coefficient) AND variation (e.g. standard deviation) or associated estimates of uncertainty (e.g. confidence intervals)
☒	☐	For null hypothesis testing, the test statistic (e.g. *F*, *t*, *r*) with confidence intervals, effect sizes, degrees of freedom and *P* value noted<br>*Give P values as exact values whenever suitable.*
☒	☐	For Bayesian analysis, information on the choice of priors and Markov chain Monte Carlo settings
☒	☐	For hierarchical and complex designs, identification of the appropriate level for tests and full reporting of outcomes
☒	☐	Estimates of effect sizes (e.g. Cohen's *d*, Pearson's *r*), indicating how they were calculated

Our web collection on statistics for biologists contains articles on many of the points above.

Software and code

Policy information about availability of computer code

Data collection	Cell Ranger v6.0.1 developed by 10x Genomics was used to obtain single cell sequencing data from fastq files after sequencing with Novaseq 6000 (Illumina).
Data analysis	Cell Ranger v6.0.1 developed by 10x Genomics was used to initially align single cell data from sequencing to reference genome of Drosophila melanogaster. SoupX v1.6.1 was used to remove ambient RNA from the single cell data. Single cell data was finally analyzed with Seurat v4.1.1 and Monocle3 v1.0.0.

For manuscripts utilizing custom algorithms or software that are central to the research but not yet described in published literature, software must be made available to editors and reviewers. We strongly encourage code deposition in a community repository (e.g. GitHub). See the Nature Portfolio guidelines for submitting code & software for further information.

Data

Policy information about availability of data

All manuscripts must include a data availability statement. This statement should provide the following information, where applicable:

- Accession codes, unique identifiers, or web links for publicly available datasets
- A description of any restrictions on data availability
- For clinical datasets or third party data, please ensure that the statement adheres to our policy

All single cell data was deposited to GEO (accession number GSE214510, reviewer token: chyxmcaqhfqnbyv).

Human research participants

Policy information about studies involving human research participants and Sex and Gender in Research.

Reporting on sex and gender	N/A
Population characteristics	N/A
Recruitment	N/A
Ethics oversight	N/A

Note that full information on the approval of the study protocol must also be provided in the manuscript.

Field-specific reporting

Please select the one below that is the best fit for your research. If you are not sure, read the appropriate sections before making your selection.

☒ Life sciences ☐ Behavioural & social sciences ☐ Ecological, evolutionary & environmental sciences

For a reference copy of the document with all sections, see nature.com/documents/nr-reporting-summary-flat.pdf

Life sciences study design

All studies must disclose on these points even when the disclosure is negative.

Sample size	40 adult eyes from Canton S Drosophila melanogaster animals were dissected for each time point for single cell sequencing. The number of eyes was empirically determined to yield enough cells for a single cell sequencing experiment with a targeted cell recovery of 10,000 cells.
Data exclusions	During the Seurat analyses, we filtered out the non-eye cells (e.g. brain cells). The exclusion was performed by removing all cells that express brain and/or glial cell marker genes. The filtered cells are still present in the raw sequencing files uploaded to GEO.
Replication	Each single cell sequencing experiment was performed from at least 20 animals. Each cell type was represented by a number of different individual cells.
Randomization	No randomization was performed. The Canton S stock is an isogenized Drosophila melanogaster line. Randomization is not required here.
Blinding	Blinding is not needed. We need to know the fly stock upon dissection.

Reporting for specific materials, systems and methods

We require information from authors about some types of materials, experimental systems and methods used in many studies. Here, indicate whether each material, system or method listed is relevant to your study. If you are not sure if a list item applies to your research, read the appropriate section before selecting a response.

Materials & experimental systems

n/a	Involved in the study	
☐	☒	Antibodies
☒	☐	Eukaryotic cell lines
☒	☐	Palaeontology and archaeology
☐	☒	Animals and other organisms
☒	☐	Clinical data
☒	☐	Dual use research of concern

Methods

n/a	Involved in the study	
☒	☐	ChIP-seq
☒	☐	Flow cytometry
☒	☐	MRI-based neuroimaging

Antibodies

Antibodies used	rat anti-Elav (RRID:AB_528218, 1/1,000), mouse anti-Pros (RRID:AB_528440, 1/100), mouse anti-Ct (RRID:AB_528186, 1/100), chicken anti-GFP (RRID:AB_300798, 1/1,000), rabbit anti-mCherry (RRID:AB_2889995, 1/2,000), mouse anti-Rh3 (gift from Dr. Steven Britt, clone 2E1 1/100), mouse anti-Rh5 (gift from Dr. Steven Britt, clone 7F1, 1/10), guinea pig anti-Rh6 (gift from Dr. Claude Desplan, 1/1,000). Secondary antibodies were used at a concentration of 1/500: Cy5 anti-rat (RRID:AB_2340672), Cy5 anti-guinea pig

(RRID:AB_2340460), Alexa 488 anti-rat (RRID:AB_141709), Alexa 647 anti-mouse (RRID:AB_162542), Alexa 488 anti-chicken (RRID:AB_2762843), Alexa 568 anti-rabbit (RRID:AB_2534017), Alexa 546 anti-rabbit (RRID:AB_2534016).

Validation	Validation data of primary antibodies are provided in the references cited in the manuscript.

Animals and other research organisms

Policy information about studies involving animals; ARRIVE guidelines recommended for reporting animal research, and Sex and Gender in Research

Laboratory animals	1-day, 3-day and 7-day post eclosion Canton S Drosophila melanogaster adult animals were used in this study.
Wild animals	This study did not involve wild animals
Reporting on sex	Male and female 1-day post eclosion adult animals were used in this study. Same number of adult eyes were dissected for male and female single cell sequencing samples. There were very few transcriptomic differences between male and female adult eyes. Adult animals were sexed with standard techniques (e.g. sex combs and male genitalia only present in males, female genitalia only in females).
Field-collected samples	This study did not involve samples collected from the field.
Ethics oversight	No ethical guidance or approval was required as the animals were fruit flies, Drosophila melanogaster.

Note that full information on the approval of the study protocol must also be provided in the manuscript.
